# The haematological, proinflammatory cytokines and IgG changes during an ovine experimental theileriosis

**DOI:** 10.4102/ojvr.v86i1.1629

**Published:** 2019-02-04

**Authors:** Gholamreza Razmi, Saeed Yaghfoori, Mehrdad Mohri, Alirez Haghparast, Shahin Tajeri

**Affiliations:** 1 Department of Pathobiology, Ferdowsi University of Mashhad, Islamic Republic of Iran; 2 Department of Clinical Sciences, Ferdowsi University of Mashhad, Islamic Republic of Iran

## Abstract

Malignant ovine theileriosis is caused by *Theileria lestoquardi*, which is highly pathogenic in sheep. Theileriosis involves different organs in ruminants. Little is known about the role of proinflammatory cytokines in the pathogenesis of *T. lestoquardi* infection. The aim of this study was to measure concentration changes of proinflammatory cytokines and immunoglobulin G (IgG) during an ovine experimental theileriosis and correlate it with clinical and haematological parameters. During an experimental study, seven healthy Baluchi sheep (four females and three males) about 6–8 months old were infected with *T. lestoquardi* by feeding of infected unfed ticks on the sheep’s ears. The infected sheep were clinically examined during the study and blood samples were collected on days 0, 2, 5, 7, 10, 12, 14, 17 and 21. The haematological parameters were analysed by an automatic veterinary haematology cell counter and the inflammatory cytokines interleukin-6 (IL-6), tumour necrosis factor-α (TNF-α), interferon-γ (IFN-γ) and IgG were measured by enzyme-linked immunosorbent assay. All infected sheep had temperatures above 40 °C on days 3–4 post infection (PI). The maximum temperature was noted on day 7, and it remained high until day 21. The parasitaemia of *T. lestoquardi* infection increased from 0.01% (day 7 PI) to 3.3% (day 21 PI). The mean white blood cell (WBC), red blood cell (RBC), lymphocyte, neutrophil and platelet values slightly increased on day 2 PI and decreased by day 17 and day 21 PI. The percentage parasitaemia and fever had a negative correlation with the numbers of WBCs, RBCs, lymphocytes, neutrophils and platelets. The serum concentration of IL-6, TNF-α and IFN-γ cytokines increased and peaked on day 12 and thereafter decreased to levels lower than 0. Out of all tested cytokines, the concentration of IL-6 was significantly higher, as early as day 2 PI. No significant changes were observed for the IgG levels during the course of disease. A significant and strong correlation was observed between IL-6, TNF-α and IFN-γ values and a moderate correlation between IL-6 and the numbers of lymphocytes in the present study. A strong correlation was determined between the percentage parasitaemia and haematological parameters in *T. lestoquardi*-infected sheep. In addition, preliminary results indicate that the measurement of the serum concentrations of IL-6 in combination with haematological parameters could be considered a good marker to estimate the pathogenicity of *T. lestoquardi* strain.

## Introduction

Ovine malignant theileriosis is an important tick-borne disease with high mortality rates. The disease is prevalent in the Middle East, East and North Africa, India, China, Central Asia and Eastern and Southern Europe (Ahmed et al. 2011; Al-Hamidhi et al. 2016; El Imam & Taha 2015). The agent of disease is *T. lestoquardi*, which is transmitted by *Hyalomma anatolicum* ticks. The life cycle of *T. lestoquardi* is similar to that of *T. annulata* (Ahmad et al. 2011; Morrison 2015). Briefly, when the ticks suck blood, the sporozoites are inoculated into blood and quickly enter monocytes and lymphocytes of associated lymph nodes near the tick bite. The sporozoites transform to trophozoites and develop to macroschizonts. The macroschizont development causes transformation and proliferation of the infected and non-infected lymphocytes and monocytes. Later, the macroschizonts develop into microschizonts that produce many merozoites in infected lymphocytes or monocytes. The merozoites are released after lymphocyte disruption and enter the erythrocytes and transform to piroplasms with ring, dot and rod forms (Ahmad et al. 2011; Morrison 2015). The pathogenicity of *Theileria* species is largely related to the ability of *Theileria* schizonts to induce high proliferation of mononuclear leucocytes and the capacity to metastasise and multiply in non-lymphoid as well as lymphoid tissues (Dobbelaere & Küenz 2004; Tretina et al. 2015). Some studies have shown that the pathogenesis of acute theileriosis could be related to the high-level production of proinflammatory cytokines during the course of disease in *T. annulata*-infected cattle (Glass et al. 2000; Graham et al. 2001) and *T. parava*-infected cattle (Yamada et al. 2009).

So far, the role of proinflammatory cytokines in the immunopathogenesis of *T. lestoquardi* infection in sheep has been studied less than that of *T. annulata* and *T. parva* infection in cattle. The aim was to measure haematological parameters and proinflammatory cytokines (IL-6, TNF-α, IFN-γ) and IgG levels and to determine the correlation of the proinflammatory cytokine levels with haematological parameters during an ovine experimental theileriosis.

## Methods

### Experimental transmission

In this study, seven Baluchi sheep (4 females, 3 males) aged between 6 and 8 months were bought from a farm which had no history of theileriosis. The sheep were experimentally infected with *T. lestoquardi* as performed previously (Yaghfoori et al. 2016, 2017). Briefly, infected adult *H. anatolicum* with *T. lestoquardi* were prepared at the Parasitology Department, Faculty of Veterinary Medicine of the Ferdowsi University of Mashhad, Iran. For experimental transmission of *T. lestoquardi*, adult ticks of *H. anatolicum* (8 males, 22 females) were placed in cotton bags on the ears of each sheep. All sheep were clinically examined on days 0, 2, 5, 7, 10, 12, 14, 17 and 21, and the clinical signs recorded. The blood and lymph node smears were simultaneously prepared and thereafter blood samples (10 mL) were taken from the jugular vein into serum and ethylenediaminetetraacetic acid (EDTA) tubes. The blood and lymph node smears were stained using the Giemsa method. The blood in serum tubes were centrifuged at ×1800 g rpm for 10 min and the serum samples were transferred to plain tubes and kept at -80 °C until the experiment was performed. The EDTA blood samples were kept at 4 °C until cell blood count (CBC) examination was performed.

### Microscopic examination

The blood and lymph node smears were stained using the Giemsa method. The stained smears were examined for detection of trophozoites and schizonts of *T. lestoqaurdi* using a light microscope at ×1000 magnification. The parasitaemia of *T. lestoquardi* infection was determined by counting parasites in 100 microscopic fields in the blood smears (Razmi et al. 2003).

### Cell blood count determination

Total cell counts and differential WBC counts were measured on days 0, 2, 5, 7, 10, 12, 14, 17 and 21 in all sheep with an automatic veterinary haematology cell counter (Nihon Kohden, Celltac α, NEK–6450 K, Tokyo, Japan).

### Sampling for cytokine measurement

Commercially available enzyme-linked immunosorbent assay (ELISA) kits (Biotechnology Laboratory, China), including IL-6, TNF-α, IFNγ and IgG, were used to measure respective cytokine and antibody levels according to the manufacturer’s instructions. Briefly, flat-bottomed 96-well plates were coated with cytokine-specific monoclonal antibodies labelled with biotin. A volume of 40 *μ*L serum sample, 10 *µ*L of anticytokine antibody and 50 *μ*L of streptavidin-HRP were added to each well and incubated at 37 °C for 60 min. Each well was washed with washing solution, then 50 *μ*L chromogenic solution A and 50 *μ*L chromogen solution B were added and incubated at 37 °C for 10 min. The reaction was stopped by adding 50 *μ*L stop solution and optical density (OD) was read at 450 nanometre (nm) in an ELISA plate reader (ELx800 Absorbance Reader, BioTek, Winooski, VT). Cytokine concentrations (ng/L) were determined by using a standard curve. Detection limits of IL-6, TNF-α, IFNγ and IgG were 2 ng/L – 600 ng/L, 5 ng/L – 400 ng/L, 5 ng/L – 300 ng/L and 0.02 mg/mL – 20 mg/mL, respectively.

### Statistical analysis

Results were shown as mean ± SEM. The Wilcoxon test was used to compare the baseline and next values from the same group of animals. The correlations between different variables were analysed using the Spearman’s test (SPSS software version 22). The strength of the correlation between the paired variables is shown as *r*_*s*_ value and is by design constrained as follows: -1 ≤ *r*_*s*_ ≤ 1. The strength of the correlation is ‘very weak’ if *r*_*s*_ values are between 0.00 and 0.19, ‘weak’ if *r*_*s*_ values are between 0.20 and 0.39, ‘moderate’ if *r*_*s*_ values are between 0.40 and 0.59, ‘strong’ if *r*_*s*_ values are between 0.60 and 0.79 and ‘very strong’ if *r*_*s*_ values are between 0.80 and 1.0. A ‘positive correlation’ is a relationship between two variables in which both variables move in tandem. A ‘negative correlation’ is a relationship between two variables that move in opposite directions. The statistical analysis and the tables and chats draws were carried out using SPSS software (version 21). A correlation *p* < 0.05 was considered statistically significant.

### Ethical considerations

The experiment was performed according to the internal regulations declared by the Animals Support Association, Ferdowsi University of Mashhad, Iran.

## Results

**FIGURE 1:**
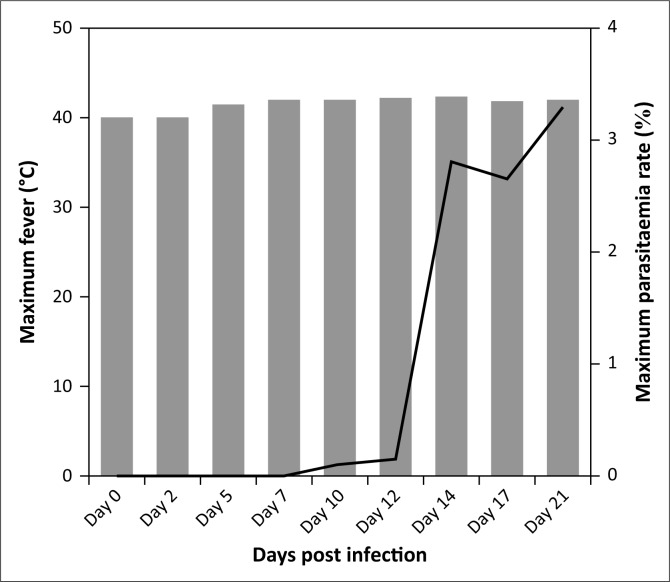
Change in body temperature (bar) and percentage of parasitaemia (line) during experimental *T. lestoquardi* infection in sheep.

In this study, all sheep were infected with *T. lestoquardi* and showed clinical signs, including fever, emaciation, lymph node enlargement, pulmonary and cardiac dysfunction, anaemia, icterus and death. Six infected animals developed acute theileriosis and were euthanised on day 21 PI. One infected animal suddenly died on day 14 PI before it could be euthanised. The body temperature increased above 40 °C on days 3–4 PI and reached a maximum on day 21 PI (Figure 1). Pre-peripheral lymph node enlargement was detected on day 4 PI. Schizonts of *T. lestoqaurdi* were microscopically detected on day 3 PI and piroplasm formed on day 5 PI. The parasitaemia of *T. lestoquardi* infection was first detected (0.01%) on day 5 PI and reached its highest rate (3.3%) on day 21 PI in infected sheep (Figure 1). The mean WBC, RBC, lymphocyte, neutrophil and platelet values slightly increased on day 2 PI and thereafter decreased until day 21 PI (*p* < 0.05) (Table 1). The levels of IL-6 were markedly elevated on days 2 PI (*p* < 0.01) and 12 PI, in comparison to the other cytokines, and decreased to levels lower than on day 0 on days 17 and 21 PI (*p* < 0.05) (Table 1). Similar but lower peak levels of TNF-α and INF-γ were also observed on day 12 PI which decreased to the lowest level on day 17 and day 21 PI (*p* < 0.05) (Table 1). The mean level of antibody IgG changes was not significant during the course of the study (Table 1). The serum concentrations of IL-6 correlated strongly and significantly with the level of TNF-α and IFN-γ in serum samples (*p* < 0.01). The correlation between IL-6 and the numbers of lymphocytes was moderate (*p* < 0.05) (Table 2). The percentage parasitaemia and fever had a strong and significant negative correlation with the numbers of neutrophils, WBC, RBC and platelets (Table 2).

**TABLE 1:** The mean levels of cell blood count, cytokine and antibody concentrations in sheep infected with *T. lestoquardi* during an experimental study.

Day	Cell blood count Mean ± SD					Cytokine Mean ± SD			Antibody Mean ± SD†
	White blood cells (10^3^/mL)	Lymphocytes (10^3^/mL)	Neutrophils (10^3^/mL)	Red blood cells (10^6^/mL)	Platelet (10^6^/mL)	IL-6 (ng/L)	TNF-*α* (ng/L)	INF-*γ* (ng/L)	
0	12.4 ± 3.9	6.2 ± 2.7	6.0 ± 1.5	10.8 ± 0.4	375 ± 45	143.8 ± 65	137.7 ± 109	57.0 ± 37	2.3 ± 0.8
2	15.9 ± 4.7	7.56 ± 2.7	8.1 ± 3.1*	10.8 ± 0.7*	312 ± 43	217.7 ± 176*	141.9 ± 125	58.1 ± 28	-
5	11.90 ± 1.1	6.3 ± 0.8	5.4 ± 1.2	10.1 ± 0.5*	335 ± 48	212.1 ± 173	143.9 ± 147	59.3 ± 42	2.3 ± 0.7
7	13.6 ± 2.2	6.9 ± 1.4	6.6 ± 1.9	9.7 ± 0.8*	296 ± 36*	202.4 ± 142	142.3 ± 164	52.6 ± 29	-
10	8.1 ± 1.8*	3.9 ± 1.0*	3.5 ± 0.8*	8.4 ± 0.4*	224 ± 18*	229.4 ± 161	153.6 ± 150	59.7 ± 28	2.8 ± 1.0
12	5.4 ± 1.2*	3.0 ± 0.6*	2.1 ± 0.8*	7.9 ± 0.7*	134 ± 35*	335 ± 283	213.3 ± 233	78.4 ± 60	-
14	4.5 ± 1.3*	2.9 ± 0.6*	1.6 ± 0.4*	7.5 ± 0.5*	234 ± 84*	248.6 ± 184	145.0 ± 123	55.3 ± 33	-
17	4.2 ± 1.2*	2.6 ± 0.9*	1.5 ± 0.4*	6.1 ± 1.2*	101 ± 56*	98.7 ± 21*	62.2 ± 10*	30.5 ± 4*	2.6 ± 0.8
21	4.8 ± 1.4*	2.7 ± 1.2*	1.7 ± 0.4*	6.1 ± 2.1*	220 ± 54*	93.7 ± 16*	62.7 ± 24*	32.1 ± 9*	2.7 ± 1.1

SD, standard deviation; IL-6, interleukin-6; TNF-*α*, tumour necrosis factor-α; INF-γ, interferon-γ.

* , Significant difference with first sampling time at *p* < 0.05.

† , IgG (mg/mL).

**TABLE 2:** Correlation between the mean levels of proinflammatory cytokines, immunoglobulin G, cell blood count, percentage parasitaemia and fever.

Spearman’s rho	IL-6 (ng/L)	TNF-*α* (ng/L)	IFN-*γ* (ng/L)	Lymphocytes (1000/mL)	WBCs (1000/mL)	Neutrophils (1000/mL)	RBCs (1000000/mL)	Platelet (1000/*µ*L)	Parasitaemia rate (%)	Fever (°C)
**IL-6 (ng/L)**										
Correlation coefficient	1.000	0.709**	0.586**	0.277*	0.192	0.223	0.209	0.120	−0.179	0.034
Sig. (2-tailed)	-	0.000	0.000	0.032	0.141	0.086	0.113	0.367	0.170	0.796
N	61	61	61	60	60	60	59	59	60	60
**TNF-α (ng/L)**										
Correlation coefficient	0.709**	1.000	0.747**	0.241	0.189	0.225	0.366**	0.078	−0.246	−0.162
Sig. (2-tailed)	0.000	-	0.000	0.063	0.148	0.085	0.004	0.557	0.058	0.217
N	61	61	61	60	60	60	59	59	60	60
**IFN-*γ* (ng/L)**										
Correlation coefficient	0.586**	0.747**	1.000	0.206	0.119	0.180	0.216	−0.003	−0.137	−0.105
Sig. (2-tailed)	0.000	0.000	-	0.114	0.363	0.168	0.101	0.982	0.295	0.423
N	61	61	61	60	60	60	59	59	60	60
**Lymphocytes (1000/mL)**										
Correlation coefficient	0.277*	0.241	0.206	1.000	0.921**	0.825**	0.622**	0.590**	−0.616**	−0.386**
Sig. (2-tailed)	0.032	0.063	0.114	-	0.000	0.000	0.000	0.000	0.000	0.002
N	60	60	60	60	60	60	59	59	60	60
**WBCs (1000/mL)**										
Correlation coefficient	0.192	0.189	0.119	0.921**	1.000	0.940**	0.773**	0.621**	−0.753**	−0.469**
Sig. (2-tailed)	0.141	0.148	0.363	0.000	-	0.000	0.000	0.000	0.000	0.000
N	60	60	60	60	60	60	59	59	60	60
**Neutrophils (1000/mL)**										
Correlation coefficient	0.223	0.225	0.180	0.825**	0.940**	1.000	0.809**	0.549**	−0.798**	−0.446**
Sig. (2-tailed)	0.086	0.085	0.168	0.000	0.000	-	0.000	0.000	0.000	0.000
N	60	60	60	60	60	60	59	59	60	60
**RBCs (1000000/mL)**										
Correlation coefficient	0.209	0.366**	0.216	0.622**	0.773**	0.809**	1.000	0.521**	−0.874**	−0.676**
Sig. (2-tailed)	0.113	0.004	0.101	0.000	0.000	0.000	-	0.000	0.000	0.000
N	59	59	59	59	59	59	59	59	59	59
**Platelet**										
Correlation coefficient	0.120	0.078	−0.003	0.590**	0.621**	0.549**	0.521**	1.000	−0.497**	−0.644**
Sig. (2-tailed)	0.367	0.557	0.982	0.000	0.000	0.000	0.000	-	0.000	0.000
N	59	59	59	59	59	59	59	59	59	59
**Parasitaemia rate (%)**										
Correlation coefficient	−0.179	−0.246	−0.137	−0.616**	−0.753**	−0.798**	−0.874**	−0.497**	1.000	0.591**
Sig. (2-tailed)	0.170	0.058	0.295	0.000	0.000	0.000	0.000	0.000	-	0.000
N	60	60	60	60	60	60	59	59	60	60
**Fever (°C)**										
Correlation coefficient	0.034	−0.162	−0.105	−0.386**	−0.469**	−0.446**	−0.676**	−0.644**	0.591**	1.000
Sig. (2-tailed)	0.796	0.217	0.423	0.002	0.000	0.000	0.000	0.000	0.000	-
N	60	60	60	60	60	60	59	59	60	60

IL-6, interleukin-6; TNF-*α*, tumour necrosis factor-α; IFN-*γ*, interferon-*γ*; WBCs, white blood cell; RBCs, red blood cell.

* , Correlation is significant at the 0.05 level (2-tailed);

** , Correlation is significant at the 0.01 level (2-tailed).

## Discussion

There is little information on the mechanism involved in the immunopathogenesis of *T. lestoquardi* infection despite its pathogenic nature (Ahmad et al. 2011). In the present study, the haematological, proinflammatory cytokines and IgG parameters were analysed during disease.

During the course of infection, all animals showed a slight leukocytosis at the onset of *T. lestoquardi* infection, and then the RBC, WBC, neutrophil, lymphocyte and platelet numbers regularly decreased with increased percentage parasitaemia. These results agreed with the results of other authors (Col & Uslu 2006; Elsadig et al. 2013; Fry et al. 2016; Hasanpour et al. 2008; Leemans et al. 1999; Nazifi et al. 2011; Omer et al. 2002; Sandhu et al. 1998) who reported anaemia, leukopenia, lymphopenia and thrombocytopenia in bovine and ovine theileriosis. The mechanism of anaemia is related to RBC lysis owing to activation of a complement system, erythrophagocytosis and increase in proinflammatory cytokine levels (Graham et al. 2001; Forsyth et al. 1999). In addition, RBC lysis may be the consequence of the damages caused to the RBC membrane because of oxidative stress (Asri Rezaei & Dalir-Naghadeh 2006; Nazifi et al. 2011, 2013; Razavi et al. 2011). A slight leukocytosis at the onset of infection could be attributed to proliferation of lymphocytes in the lymphoid organs as a defensive response to the *Theileria* infection. Leukopenia in the terminal stage could have resulted from large-scale destruction of lymphocytes by schizogony in lymphoid organs and infiltration of these cells into various organs (Sandhu et al. 1998). The thrombocytopenia with decreased RBCs and WBCs could also be related to increased TNF-α production (Forsyth et al. 1999) or suppression of the bone marrow by the parasite and its products (Abd Ellah 2015; Mbassa et al. 1994). In this study, the levels of IL-6, TNF-α and IFN-γ were increased from the onset to the middle course of disease and decreased in the terminal stage of disease in infected sheep. *In vitro* studies have reported high mRNA expression levels of proinflammatory cytokines (IL-1*β*, IL-6 and TNF-α) in leucocytes infected with *T. annulata* or *T. parva* schizonts (Brown et al. 1995; Collins et al. 1996; McGuire et al. 2004; Mckeever, Nyanjui & Ballingall 1997).

Some studies have shown a remarkable elevation in the levels of IL-1*β*, IL-6, IFN-γ and TNF-α cytokines in *T. annulata*-infected cattle (El-Sebaei, El-Ashker & El-Boshy 2014; Razavi et al. 2010; Nazifi et al. 2010) and *T. lestoquard*-infected sheep (Razavi et al. 2015) in farm conditions. Despite the high correlation observed between IL-6 levels with TNF-α and INF-γ levels during the course of disease, only IL-6 was significantly increased. The sources of IL-6 are monocytes and macrophages. The crucial role of IL-6 is B- and T-cell activation and the induction of the acute phase response (Hunter & Jones 2015; Tanaka & Kishimoto 2012). During acute inflammation, IL-6 is the first responding cell factor which will induce the generation of C-reactive protein (CRP), serum amyloid A, fibrinogen and hepcidin in hepatocytes (Reinhart et al. 2012; Tanaka, Narazaki & Kishimoto 2014). IL-6 has a longer plasma half-life than TNF-α or IL-1β and is more reliably measurable in plasma than the other two cytokines. It also has other potential clinical uses, such as diagnosis and management of autoimmune rheumatic disorders. Its major role as a biomarker of sepsis appears to be prognostic, and not diagnostic (Faix 2013; Reinhart et al. 2012). A correlation between high levels of IL-6 with mortality rate has been reported in systemic bacterial infection in humans and dogs (Nijsten et al. 1978; Oda et al. 2005; Rau et al. 2007), in malaria (Day et al. 1999; Mbengue et al. 2015) and canine babesiosis (Goddard et al. 2016). The role of IL-6 in immunopathogenesis of theileriosis is unclear. Some studies have shown that high IL-6 expression was related to virulence of *Theileria* strains and host susceptibility (McKeever, Nyanjui & Ballingall 1997; Yamada et al. 2009), and non-detectable IL-6 expression correlated with resistance of sheep to *T. annulata* (Schnittger et al. 2000) and African buffaloes to *T. parva* (Okagawa et al. 2012).

## Conclusion

Based on the results, the WBC, RBC, lymphocyte, neutrophil and platelet values decreased with increased percentage parasitaemia and temperature during the course of *T. lestoquardi* infection in sheep. In addition, a high correlation was observed between serum levels of IL-6 with levels of TNF-α and INF-γ. Preliminary results indicate that the measurement of the serum concentrations of IL-6 cytokine in combination with haematological parameters could be considered a good marker to estimate the pathogenicity of the *T. lestoquardi* strain.
